# Financial and Socio-Economic Effects of Investment in the Context of Dog Population Management

**DOI:** 10.3390/ani12223176

**Published:** 2022-11-17

**Authors:** Jasmina Ćetković, Miloš Žarković, Miloš Knežević, Meri Cvetkovska, Radoje Vujadinović, Snežana Rutešić, Željka Beljkaš, Marija Grujić, Bojan Adžić

**Affiliations:** 1 Faculty of Economics, University of Montenegro, 81000 Podgorica, Montenegro; 2 Faculty of Civil Engineering, University of Montenegro, 81000 Podgorica, Montenegro; 3 Faculty of Civil Engineering, Ss. Cyril and Methodius University, 1000 Skopje, North Macedonia; 4 Faculty of Mechanical Engineering, University of Montenegro, 81000 Podgorica, Montenegro; 5 Faculty of Civil Engineering, University of Belgrade, 11000 Belgrade, Serbia; 6 Diagnostic Veterinary Laboratory, 81000 Podgorica, Montenegro

**Keywords:** welfare of animals, stray dogs, shelter, cost–benefit analysis, socio-economic benefits

## Abstract

**Simple Summary:**

The problem of stray dogs, especially in urban areas, is a challenge for the modern world. We investigated this problem in a sample from the Vardar Planning Region in North Macedonia, where there has been an increase in the number of stray dogs. The aim of this paper is to evaluate the financial and socio-economic justification for the construction of a new shelter for stray dogs in this region, as existing capacities are not sufficient. The analysis confirmed that the socio-economic benefits from this project are multiple, including savings in stray dog bite costs, savings in the cost of traffic accidents caused by stray dogs, and savings in the treatment of diseases caused by stray dogs. The results of the economic analysis show that the Economic Net Present Value is positive and amounts to EUR 789,916. The Economic Internal Rate of Return is 25.94% and the Economic Benefit–Cost Ratio amounts to 1.90, i.e., greater than 1. Quantifying the additional socio-economic benefits of such projects may be a challenge for future research. The results of our analysis can benefit the decision-makers who will be involved in the implementation of this and other similar projects, which represents a practical contribution of our paper to this field.

**Abstract:**

The modern world faces serious challenges associated with the presence of stray dogs on the streets, especially in urban areas. Vardar Planning Region in North Macedonia, which consists of nine municipalities, experiences such challenges. According to current reports, the number of stray dogs on the streets of cities in this region has increased, which has resulted in an increase in the number of dog attacks on residents. As the existing capacities are small in the registered shelters, we considered the possibility of building a new shelter for stray dogs to meet the needs of this region. The goal of our paper is the evaluation of the financial and socio-economic justifications for the construction of a shelter for stray dogs in the Vardar Planning Region (VPR). The results of the financial justification analysis show that the project does not provide satisfactory financial results. Namely, the Financial Net Present Value (FNPV) is negative, with a value of EUR 75,291. The Financial Internal Rate of Return (FIRR) is 0.57%, lower than the discount rate, which is not acceptable for a private investor. The Financial Benefit–Cost Ratio (FB/CR) of this project is 0.925, suggesting that the total discounted costs are greater than the total discounted revenues. On the other hand, the expected socio-economic benefits from this project are multifaceted, including savings in stray dog bite costs, savings in the cost of traffic accidents caused by stray dogs, and savings in the treatment of diseases caused by stray dogs. The results of the economic analysis show that this investment has full socio-economic justification and that it should be implemented. The Economic Net Present Value (ENPV) is positive and amounts to EUR 789,916. The Economic Internal Rate of Return (EIRR) is 25.94% and the Economic Benefit–Cost Ratio (EB/CR) amounts to 1.90, i.e., greater than 1. The results of the sensitivity analysis also confirm the justification for the realization of this project.

## 1. Introduction

In order to improve the welfare and health of stray dogs and reduce the problems associated with them, a multifaceted, humane, and ethical concept called Dog Population Management (DPM) was developed. DPM interventions can be put into practice in many locations, but DPM design must always match local conditions [1].

In some countries, owners bring their dogs to shelters when they cannot take care of them (relinquishment), while in other countries this practice is less common, and dogs are instead abandoned in the street (abandonment). Studies from the 1990s found that illness and old age are two important reasons for relinquishment [2]. Salman et al. [2] determined that the change in residence of the owner is the most common reason for relinquishment. Other recognized reasons for relinquishment are: the costs of keeping pets, a lack of time for the pet, inadequate accommodation, many pets in the household, etc. In some studies from the 1990s, other reasons for the relinquishment of animals were recognized as personal problems: conflict between the pet and child, death of the owner, illness, pregnancy, lack of time, divorce, and the desire to travel [3,4]. Additionally, Kidd et al. [5] showed that unrealistic expectations for pet behavior are identified as a risk factor for relinquishment, while certain studies concluded that cross-cultural differences among countries influence how significant this factor will be [6]. Later, Neidhart and Boyd [7] also tried to recognize the factors of relinquishment, such as initial dog cost, expectations for dog behavior, and duration of ownership, among others.

Stray dogs represent a problem of modern society related to public health and safety, but also to the welfare of animals. At the same time, animal welfare primarily depends on human behaviors, influenced by attitudes [8]. According to the World Organization for Animal Health (WOAH), the term stray dogs refers “to any dog not under direct control by a person or not prevented from roaming” [9]. This population includes three types of dogs: free-roaming owned dogs, free-roaming dogs with no owner, and feral dogs. According to Hughes and Macdonald [10], the free-roaming dog category includes about 75% of dogs across the world, which is a high percentage, while their number varies from country to country, depending on various factors—economic/social/cultural/demographical, etc. [11]. Where the density of stray dogs is at a high level [12], this category represents a serious problem for the public health of the population, being associated with the occurrence of various diseases [13,14], and also with the endangered welfare of other animals. Knobel et al. [15] estimated that the rabies virus alone, which in 99% of cases is transmitted to humans by dogs, causes about 55 thousand deaths per year globally. Later, Hampson et al. [16] estimated that this was close to 60 thousand deaths per year, representing an economic cost of about USD 8.6 billion. In addition to those previously mentioned, many diseases, such as leishmaniasis, echinococcosis, and toxocariasis, might cause serious health problems for the human population [17,18,19]. Additionally, the problems related to the transmission of various pathogens, dog bites [20,21], and traffic accidents caused by stray dogs [22] represent an additional and serious threat to the population, especially in urban areas. That is why the focus of population management is precisely on this category of dogs [9], with the aim to reduce problems related to public health and the environment, but also to improve the health of this category of dogs [23]. Stray dogs, due to poor health and social conditions, face various challenges [24,25,26]. From that mentioned above, it follows that different countries and regions focus on different objectives for dog population management [1,27], such as: reducing the number of stray dogs, improving the health of this population, protecting public health, reducing the risk for other animal species, increasing awareness for responsible ownership, etc.

An additional danger is the fact that the population of stray dogs is not easy to recognize on the street. It is important to point out that it is ineffective to focus on stray dogs without addressing the sources of these dogs. This problem was previously solved by euthanizing a large number of animals [28]. However, the perception of this problem has changed and a large number of animal protection NGOs, as well as public authorities, are investing in increasing adoption programs in shelters and minimizing euthanasia. For this reason, shelters should be used where there is a large number of stray dogs, but also a significant adoption rate. However, some studies have noted that current owners are more attached to their pets adopted from the shelter compared with relinquishers, and that they are part of the family [29,30]. Recently, Sandøe et al. [31] showed that, in general, when dogs are kept in controlled conditions, with strict regulation, the problem of stray dogs is limited and shelters can handle it well. Certain studies have observed that stray dogs make up 53–83% of the total population in shelters [32,33,34,35]. The factors that contribute to the country being free from stray dogs are primarily related to cultural and social factors [36]. As the construction of a shelter cannot solve all of the problems related to stray dogs, it is necessary to continuously work on other methods of population control, such as reproduction control, controls of commercial breeding and sale, communication strategies, education and raising awareness about responsible ownership, etc.

Although the level of prosperity of the country has an impact on the reduction of the number of problems related to stray dogs, Reece et al. [37] showed that CNVR (Collect, Neuter, Vaccinate, and Return) is a proven method that has been justified in some countries, which helps reduce the stray population, regardless of the prosperity of the country. Each year, millions of dogs enter animal shelters, while in some countries this is the most common method of controlling stray dogs. Some studies have pointed to the fact that there are more dogs entering shelters than leaving them, which can open up them up to the problem of overcrowding [38,39]. In principle, building shelters is expensive and requires serious planning and strong organization. It is a fact that the investments required for the construction of shelters are not small. On the other hand, there are serious problems in quite a few countries that have manifested through a large number of complaints from citizens. That is why it is necessary for institutions dealing with the problems related to stray dogs to use a localized database, so that the problem can be continuously monitored [40]. Shelters should provide continuous care for stray dogs, although this is not a fundamental service of DPM. This concept cannot be humane if it advocates the indiscriminate killing of stray dogs, or the use of euthanasia as the only measure to manage the dog population. The construction of shelters cannot completely solve all of the problems related to stray dogs, but at the same time the sources of future stray dogs must be monitored through various control measures. Otherwise, shelters are ineffective and will not achieve their goals. Before the decision to build shelters is made, the needs of the area must be looked at in detail. A preliminary assessment of the current situation should include existing shelters in that area, population size, the identification of the trends in the dog population that require help compared with previous years, the recognition of the area that will be serviced by the new shelter, the evaluation of the stray population in that area, the analysis of existing stray collection programs, the assessment of the financial situation in the area and the identification of funding sources (donations or local authority funding), the analysis of local attitudes to strays, etc. [41].

Rehoming, neutering, and euthanasia represent the most important policy decisions for every shelter. Shelter policies should ensure the continuous care of animals, the establishment, maintenance, and monitoring of standards, the good functioning of shelter staff, and compliance with relevant legislation. Within the shelter, the identification of abandoned animals is carried out. This includes the use of different techniques (identification tag, collars, and microchips) [42]. Additionally, sterilization programs are extended as animal control measures that limit shelter intake [43]. Interestingly, Protopopova and Gunter [44] warned about the problems related to the lives of stray dogs in some shelters, expressing concern that these animals sometimes live in poor conditions with uncontrolled reproduction and are euthanized in large numbers.

According to data from the United States collaborative initiative—Shelter Animals Count—animals leave shelters in different ways: transfer to other organizations, return to owners, adoption, natural death, or euthanasia [45]. Unfortunately, some earlier research revealed that close to 50% of dogs were euthanized instead of being adopted as initially expected [33], with the lack of shelter space being the main reason, and not behavioral or health problems. According to Greenwood [46], the rate of euthanized animals is up to 10%, with euthanasia in shelters only performed when it comes to incurable animals, or when they pose a danger to the community. However, Hawes et al. [47] have shown that euthanasia is also carried out for other reasons: costly treatment of diseases, age, breed, and other characteristics of the animals [47]. According to the study of Wenstrup and Dowidchuk [33], conducted on a sample of 42 countries and 186 shelters, analysis of the structure of the population of dogs in shelters showed that 53% were stray dogs, 43% were relinquished, 2% were other, and for 2% no answer could be found. The average retention of adopted dogs in shelters was 53 or 85 days depending on whether they were urban or rural shelters [6]. Turken et al. [48] showed that animal shelters strive to reduce the population of stray animals and the use of euthanasia through capacity expansion, adoption programs, and the merging of shelters. However, the same research found that in traditional shelters, euthanasia does not decrease with increasing demand for animals and that increasing their capacity does not necessarily decrease the number of euthanized animals.

Shelters can define different adoption strategies, and it depends on their implementation as to whether these strategies will have a positive or negative impact. One study showed that increasing the number of animals without increasing adoption fees or donations led to a faster increase in costs than total revenues [49]. This is why shelter managers are expected to separate animal-related/variable costs from fixed costs. The adoption of animals is the general goal of shelters, and shelters are expected to provide an environment that meets the animals’ welfare needs [50]. Winograd [51] emphasized that “adopting an animal means a shelter does not kill that animal”, whereby adoption frees space, allowing an additional number of animals to enter the shelter and a greater number of lives to be preserved. In order to improve the animal shelter system, it is necessary to move from a reactive to proactive approach, as well as to a comprehensive shelter orientation. The same study indicated that the rate of saving animals can be very high and that the rate of lifesaving has increased to over 90% for dogs.

Sound strategies should support adoption protocols in order to achieve the mission of the shelter and maximize the number of animal lives saved. Each shelter must identify the optimal strategy, which depends on the unique characteristics of the community. Furthermore, shelters should conduct an internal staffing assessment to define the unique skills and competencies of shelter employees and volunteers.

The aim of this paper is to evaluate the financial and socio-economic justification for the construction of a shelter in Vardar Planning Region (VPR), North Macedonia, with numerous and accumulated problems related to the care of stray dogs. It should be noted that our paper is the result of recent research that was conducted for the purposes of creating a feasibility study for a shelter construction project for stray dogs in the VPR, North Macedonia. We identified a research gap related to the deficit of relevant resources in the literature that consider the financial and socio-economic effects of the construction of such facilities. We believe that our research provides a methodological contribution in terms of quantifying and monetizing these effects, especially the socio-economic ones, which are important for the wider community. Thereby, we offer an appropriate methodological form for the assessment of such projects, with the aim of increasing the efficiency of the decision-making process for investments.

The paper is organized as follows. In the Introduction, here, we outline certain problems and aspects related to the welfare of animals, especially stray dogs. In the Current Status section, we present the current situation in the VPR, North Macedonia, regarding stray dogs, which indicates the obvious need to build a shelter, along with other population control measures, in order to bring the problem under control and begin to solve it. The Materials and Methods explains the basic methodological framework for determining the financial and socio-economic feasibility of the shelter construction project for stray dogs in North Macedonia. We emphasize that the reference European Commission methodology for cost–benefit analysis (CBA) was used for creating a feasibility study in this project. In the Results and Discussion section, we present in detail the procedure and the results obtained from the financial and socio-economic feasibility study of this project. At the end of the paper, the final conclusions are offered, alongside certain limitations, as well as possibilities for supplementing the research in the future in order to achieve improvements in this area.

## 2. Current Status: The Case of the Vardar Planning Region, North Macedonia

The World Organization for Animal Health assessment [52], founded in the Western Balkans region in the period 2015–2018, identified that the largest source of dogs on the streets is irresponsible ownership, i.e., dogs that were previously owned. This assessment, in addition to other Balkan countries, also applies to the population of stray dogs in North Macedonia. Therefore, WOAH launched the “Be his hero” campaign in these countries, which promoted the practice of responsible ownership among dog owners.

**Figure 1:**
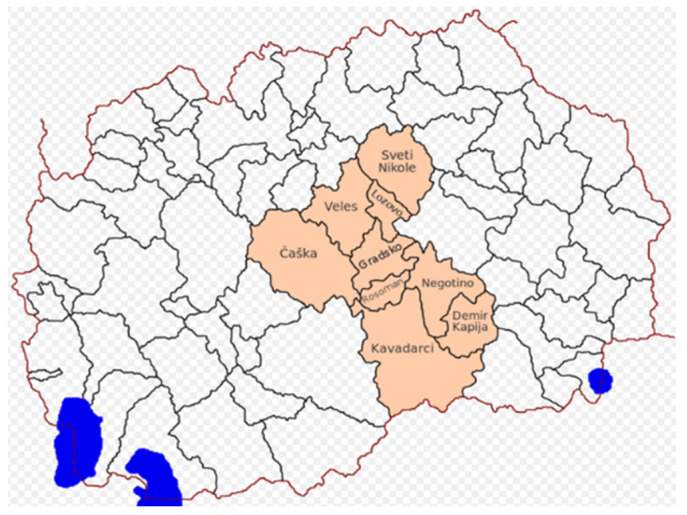
Map of the Vardar Planning Region.

The VPR region in North Macedonia faces similar challenges related to stray dogs. The VPR consists of nine municipalities, as shown in Figure 1: Veles, Negotino, Sveti Nikole, Čaška, Demir Kapija, Lozovo, Gradsko, Kavadarci, and Rosoman. The region covers an area of 4042 km^2^, and according to the results of the 2002 census, it has 154,603 inhabitants.

In the information system of the Food and Veterinary Agency in the VPR, there are a total of 10,954 registered dogs, as well as 6961 owners. Considering that the number of stray dogs in urban areas depends on the availability of food and water, the accelerated urbanization of the region has brought with it a greater availability of food for homeless animals. The sources of food are different, such as restaurant waste, garbage, food that is left for stray dogs, etc. [53]. With the irresponsible attitude of the owners who leave their dogs, there exist a large number of problems connected to stray dogs on the streets in the VPR. The significant number of stray dogs on the streets is causing a growing number of communal problems in the municipalities of VPR. Almost 40% of all citizen complaints to public utility companies relate to problems with stray dogs. Due to the increase in the number of stray dogs on the streets of VPR cities, the number of dog attacks on citizens is increasing, and thus the number of reports. In the municipality of Veles, 6–10 reports of citizens per month refer to dog bites, which generates a large outflow from the municipal treasury for fines and court costs, presenting a significant challenge for local governments. According to the data obtained from the municipality of Veles, in the last 5 years, MKD 10.6 million was spent, of which MKD 4.5 million was spent in 2021.

In the region, until 2022, no assessment of the population of stray dogs had been made. In order to make a decision on the need to build a new shelter, a feasibility study was conducted to assess the population of stray dogs in the VPR.

We decided to count the dogs using the methodology designed for counting dogs in street research, developed by the International Companion Animal Management Coalition [54]. This methodology involves determining the density of the population and its composition by the direct observation of dogs. Population density is expressed by the number of dogs per kilometer of street. The composition of the population is determined in terms of gender, age, and reproductive activity. In addition, well-being is assessed in terms of body and skin condition.

The counting was carried out by four persons over nine consecutive days, in the early morning hours in all nine municipal centers, and it was completed in March 2022. Based on the collected data, we conclude that the number of dogs that wander in the early morning hours in the cities of the Vardar region is 1231 (by municipality: Veles, 412; Sveti Nikole, 389; Kavadarci, 207; Negotino, 87; Gradsko, 21; Rosoman, 23; Demir Kapija, 66; Čaška, 21; and Lozovo, 5).

Intensive dog counting is demanding in terms of time and resources and is accomplished by the marking (photographing) of each dog. During the counting, a certain number of dogs remain unseen [55], and bearing in mind that four out of nine municipalities are rural, our estimation is that 30% of dogs are unseen during the counting. Based on this fact, we noted that in the Vardar region, that range is estimated to be from 1231 (counted dogs) to a maximum of 1600 stray dogs. The density of dogs on the streets is higher in the cities (Demir Kapija, 3.5/km; Veles, 2.44/km; Sveti Nikole, 2.2/km; Kavadarci, 2.03/km; and Negotino, 1.75/km) than in smaller places, i.e., villages (ranging from 0.57/km in Lozovo, 0.84/km in Gradsko, and 1.77/km in Rosoman to 1.81/km in Čaška). We noticed that a particularly high number of stray dogs was recorded in Demir Kapija, at 3.5/km, while dogs with ear tags were noticed only on the streets in Veles.

Although not ideal for comparison, due to different methodologies, our research on the number of stray dogs in the VPR corresponds to the number of stray dogs in the Polog region, which was measured in 2014 by the Food and Veterinary Agency of North Macedonia. Thus, we compared the estimated number of stray dogs in Veles (44,000 inhabitants) with cities of similar size in the Polog region. That number is higher than the number of stray dogs in Tetovo (224 animals) and lower than in Gostivar (420 animals).

The basic law covering the control of the dog population in Northern Macedonia is the Law on the Protection and Welfare of Animals [56,57,58]. Article 28 of this Law regulates the duties of the municipalities and the capital Skopje for the control of the population of stray dogs. This article prescribes temporary accommodation for dogs found in public urban areas in shelters, and the responsibilities of the shelters themselves (regular feeding and watering, socialization tests, castration, preventive health care and health care, microchipping, and, finally, a return to the street). The same article regulates who pays the costs for the captured dogs, the possibility of euthanasia of the animals in the case of an infectious disease, the records of the animals, etc. Article 29 of the same law regulates the manner of approval of the shelters, i.e., the competent body for the approval of the work of the shelters.

The register of approved shelters for stray dogs of the Food and Veterinary Agency has a total of 16 shelters for dogs. The data we received from the Agency show that there are currently two active shelters in the territory of VPR. One shelter is located in the territory of the municipality of Negotino, within the veterinary station Negotino, and the other is in the village of Vojnica, in the municipality of Časka. In addition to these two shelters, there are two other shelters outside the VPR, both in the municipality of Kumanovo, which control the dog population for the municipalities of Sveti Nikole and Demir Kapija. Table 1 shows the registration numbers, the owners of the shelters, and the municipality in VPR covered by the shelter.

As shown in Table 1, the following municipalities perform the control of the dog population: Sveti Nikole, Negotino, Kavadarci, Rosoman, Demir Kapija, and Veles. These municipalities adopted programs for the control of the population of stray dogs, including the program for the protection and welfare of animals—treatment of stray dogs, namely unregistered and registered dogs found in a public area without the presence of the owner. This program in Negotino, Kavadarci, and Rosoman considers the capture, neutering, vaccination and release of dogs to be implemented by the veterinary station in Negotino, which has a registered shelter and agreements with these three municipalities. Beside these three municipalities, this shelter also covers the municipality of Radoviš, which is not part of the VPR. The shelter in Časka covers the municipality of Veles, and operates in cooperation with the veterinary practice in Veles. The shelter in Negotino has 17 boxes, while the shelter in Časka is registered for 10 boxes. Both shelters were over 90% full during our visit. The shelter in Čaška also has additional unregistered capacities. Data from the Food and Veterinary Agency from the end of 2021 show that the other two shelters outside the VPR (in Kumanovo) treated only six dogs from the VPR (five from Sveti Nikole and one from Demir Kapija) in half a year, which indicates the poor enforcement of stray dog population control in the municipalities of Demir Kapija and Sveti Nikole. Our data also indicate this, recording a high population density of stray dogs in these municipalities, especially in Demir Kapija.

There are also a number of unregistered dog shelters in the region, which are funded by donations. Although, formally, six municipalities carry out programs that include releasing marked dogs (with ear rings), we found marked dogs only in the municipality of Veles (19% of counted dogs in this municipality). Besides the huge number of dogs counted on the streets, the small percentage of marked dogs (with ear rings) in the whole region suggests that programs are not conducted in the appropriate way. However, we mention that the control of the stray dog population is not the main activity of both active shelters in VPR, because the shelter in Negotino works within the veterinary station, and the shelter in Čaška is also a dog training center. While most dogs are adopted from the shelter in Čaška, which is certainly commendable, according to available data of the Agency and conversations with veterinarians, a large number of dogs in Negotino are euthanized due to not passing the socialization or disease test. The municipalities of Lozovo, Časka, and Gradsko do not control the dog population at all through dog shelters; however, the number of treated dogs from other municipalities of the VPR in the shelters is quite small.

Such small capacities (27 boxes) in the registered shelters certainly do not meet the needs in this region, which justifies the need for the construction of a shelter of larger capacity in the VPR. Namely, there is a requirement for a more effective approach in solving the problem of controlling the population of stray dogs. Solving this problem would significantly improve the health and well-being of animals, but also of humans, in terms of bites, traffic accidents and the possibility of transmitting dangerous infectious and parasitic diseases. In the medium and long term, it is necessary to work on an effective approach and strengthen control, which will lead to a reduction in costs for fines and court costs, which municipalities set aside each year. A more effective approach involves a range of measures to be taken, both locally and at the state level.

## 3. Materials and Methods

In our paper, which analyzes the justification of building a shelter for stray dogs from the Vardar region, North Macedonia, we applied cost–benefit analysis (CBA) as the basic methodological framework. Typically, CBA considers multiple costs and benefits (financial and socio-economic), where lost benefits are treated as costs, and cost savings as benefits. Initially, the financial effects of this project were assessed through a financial feasibility analysis of the project. Afterwards, we carried out an economic feasibility analysis, with an assessment of the costs and benefits of the project for the social community as a whole. We conducted both analyses using the relevant European methodology [59].

In the financial analysis of the project, in order to determine the net profit of the project, we prepared the quantification of all costs and benefits of this project. As CBA is not used exclusively for the assessment of the market, i.e., the commercial effects of the project, this methodology was used for the additional assessment of the non-measurable and indirect effects of the construction of shelters in North Macedonia. In order to monetize these effects, we use “shadow prices” as a correction of market prices. Projects of this type, such as the project to build a shelter for stray dogs, have socio-economic effects that should be quantified and monetized, as they are obvious and indisputable, in addition to their direct market and commercial effects. In addition, with such investments, socio-economic effects exceed direct market effects, and it is precisely these that influence the decision whether to implement a specific project or not. This is why it is recommended that CBA, as a suitable methodology, should be used in the process of making a decision on the implementation of projects when the focus is not on profit orientation. To this end, policy makers are suggested to use this methodology in order to achieve public interest through the implementation of such projects and achieve the optimal allocation of limited resources, which should satisfy the expectations and interests of certain subjects, and also society as a whole.

In the first part of the analysis, the financial justification of building a shelter for stray dogs was assessed. The financial analysis was conducted by taking into account the following basic assumptions:

In analysis, we used EUR and real (constant) prices;
The starting year of the analysis is 2023;
The estimated construction period of the facility is 1 year (year 2023);
The observed period of exploitation is 10 years (2024–2033);
The final year of the analysis is 2033;
The financial discount rate was set at 4%, according to the recommendations [59].

In accordance with the recommendations of the relevant European methodology [59], the analysis of the financial feasibility of the construction of a shelter for stray dogs was carried out in several steps:

Calculation of total investment costs, or total project investment costs (CAPEX), namely: land costs, construction costs, vehicle procurement costs, cost of shelter equipment purchase, cost of purchasing veterinary equipment, cost of planning and project documentation, and cost of supervision;

Defining the level of operating costs (OPEX), which for the purposes of this analysis are divided into fixed and variable costs;

The projection of the number of stray dogs in the Vardar region, whereby this projection takes into account the following assumptions: dog mortality rates (7% per year according to the average life expectancy of 14 years for certain dog breeds [60,61], dog birth rates (30% without project implementation; 15% with project implementation), and projected adoption and euthanasia rates of dogs (20% total for both categories) in the existing shelters and the new shelter. According to the stated assumptions, we provide a projection of the number of stray dogs “with” and “without” the project;

The projection of total annual operating costs was made in accordance with the assumption of the projected GDP growth rate in North Macedonia, in the period covered by our analysis;

Defining the elements for the calculation of project revenues, on the basis of which the projection of project revenues was given, for the period 2024–2033;

The evaluation of the financial justification of the project was conducted using standard dynamic indicators of the justification of the investment, namely: Financial Net Present Value (FNPV), Financial Internal Rate of Return (FIRR), and Financial Benefit–Cost Ratio (FB/CR). For the project to be financially justified, the FNPV should be greater than 0, the FIRR should be greater than the defined discount rate, and the FB/CR should be greater than 1.

Since projects of this and similar types have numerous effects of a non-market, i.e., non-commercial, nature, the procedure of their quantification and monetization is necessary. Acknowledging the fact that the market prices of input and output values do not always reflect their social value, the objective of the economic feasibility analysis of the project was to determine the socio-economic costs and benefits of the project, in order to evaluate the net contribution of the project to the social community as a whole. The basic methodological determinants for conducting this project’s economic feasibility analysis were:

CBA was performed in such a way that the basic principles and rules upon which the analysis was based were set in accordance with the methodological principles and rules of the European Commission [59] and international financial institutions;

The transformation of market into accounting (economic) prices was conducted with the help of standard conversion factors (SCF);

In order to reduce costs and benefits to the same base year, a discounting process was performed. According to the methodological recommendations from the European Commission [59], a recommended discount rate of 5% was used for countries acceding to the EU;

Within economic analysis, the following socio-economic benefits of the project were quantified and monetized: savings in the costs caused by stray dog bites, savings in the cost of traffic accidents caused by stray dogs, and savings in the treatment of diseases caused by stray dogs.

With the application of the CBA, the following indicators of project evaluation from the socio-economic aspect were determined: Economic Internal Rate of Return (EIRR), Economic Net Present Value (ENPV), and Economic Benefit–Cost Ratio (EB/CR). By comparing the value of EIRR with OCC (opportunity cost of capital) and comparing the value of ENPV with 0, the assessment of shelter construction from the socio-economic aspect was determined. Net Present Value (NPV) is an indicator that takes into account time preferences and represents the sum of net effects in the economic life of the project, discounted to the present moment, i.e., at the start of the investment. The Internal Rate of Return (IRR) is the rate at which the NPV of a project equals 0. This rate reflects the efficiency of the project, and the eligibility criterion is that it should be higher than the discount rate. The Benefit–Cost Ratio (B/CR) shows how much net benefit can be achieved per unit of cost. It is calculated as the ratio of the discounted sum of all future benefits and the discounted sum of all costs. The economic benefits of the project should be greater than the costs of the project, which is reflected in a positive Economic Net Present Value (ENPV), an Economic Benefit–Cost Ratio (EB/CR) of more than 1, and the Economic Internal Rate of Return (EIRR), which should be higher than discount rates (used to calculate NPV).

CBA in our paper concludes with sensitivity analysis. Previous indicators (ENPV, EIRR, and EB/CR) have also been subjected to a sensitivity test, given the possible deviations in the realization of economic costs for construction and economic benefits from construction. Namely, within this analysis, we varied the following key parameters: the volume of shelter work, the size of the capital investment, and the discount rate.

**Figure 2:**
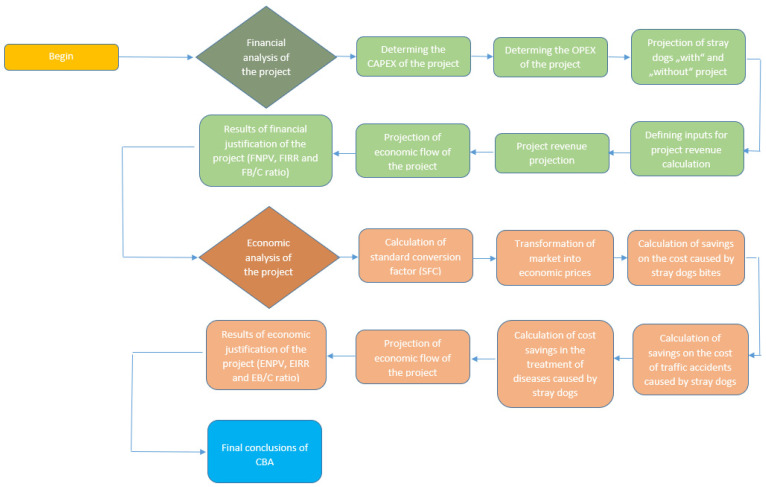
Methodological flowchart.

In the continuation, in Figure 2, we present a methodological flowchart so that readers can better follow and understand the procedure of the conducted financial and economic analysis of the justification for the construction of a shelter for stray dogs in the VPR, North Macedonia, which follows in the next section of the paper.

## 4. Results and Discussion

In the first part of this section, we present and discuss the results of the financial justification of the construction of shelters for the needs of the Vardar region. Social benefits from the project are evaluated in the second part of this section as a result of the conducted economic feasibility analysis. A sensitivity analysis is presented at the end of this section, which was intended to check the sensitivity of this project to changes in some of the key imputations from the analysis.

### 4.1. Financial Analysis

The aim of this financial analysis is to determine the investment and operating costs required for the implementation of this project, assessing its financial feasibility and sustainability. This analysis should serve as a basis for the municipalities of this region, as well as other stakeholders, to decide on how to finance investments and to cover the operational costs during the project exploitation. Financial analysis serves as the basis for the further economic evaluation of the project within the socio-economic analysis, drawing conclusions about the economic feasibility of this project.

The investment costs of this project include the following cost categories: land costs, construction costs, vehicle procurement costs, costs of shelter equipment purchase, costs of purchasing veterinary equipment, costs of planning and project documentation, and costs of supervision.

The estimated total investment costs amount to EUR 366,251, with the following structure (Table 2).

We note that re-investments in certain investment categories will appear in the financial and economic analysis, for those items whose lifespan is shorter than the analysis period. The service life of facilities and equipment is determined on the basis of the official Decree of the Republic of North Macedonia [62]. The service life of the facility is 40 years, that of vehicles is 5 years, while the service life of the equipment varies depending on the type of equipment (2, 4, 5, or 10 years), which is shown in Table 3.

The operational costs of the project include all of the potential costs necessary for the operation of this facility, after its construction. For their more adequate perception and projection, these costs are divided into fixed and variable costs.

Fixed operating costs, in the initial year of operation of the project, are set at EUR 94,070, with the following structure (Table 4).

Additionally, according to information received from representatives of municipalities in the Vardar region, as well as the Center for Development of the VPR, salary costs will be covered directly by municipalities, regardless of the future shelter’s operations, and are excluded from the shelter’s costs. The manner of covering these costs would be defined proportionally, in relation to the number of inhabitants in the municipalities of the Vardar region, in accordance with the mutual agreement. In this regard, these costs have not been further calculated in the context of this financial and economic analysis.

Considering all of the above assumptions, the variable operating costs, in the initial year of operation of the project, were set at EUR 72,000. Table 5 presents the structure of the variable operating costs.

These costs were increased in the further period of the projection with the projected GDP growth rate, in accordance with the projection of the number of dogs that will be treated. It should be additionally mentioned that the costs of the veterinary clinic during the projection were reduced for the coverage of dogs that would be sterilized—in accordance with the veterinary assumptions. The projection of the GDP growth rate is shown in Table 6.

The projection of the number of stray dogs was made on the basis of the initially determined number of stray dogs in the Vardar region and assumptions about the movement of this population in the coming period. These projections were taken into account: dog mortality rates (7% per year according to the average life expectancy of 14 years), dog birth rates (30% without project implementation, 15% with project implementation), as well as projected adoption and euthanasia rates (total of 20% for both categories) in the existing shelters in the North Macedonia and the new shelter. Data on the potential maximum number of dogs previously determined were used as the basis for the projections. Projections of the number of dogs “with project” and “without project” are shown in Table 7 and Table 8.

**Figure 3:**
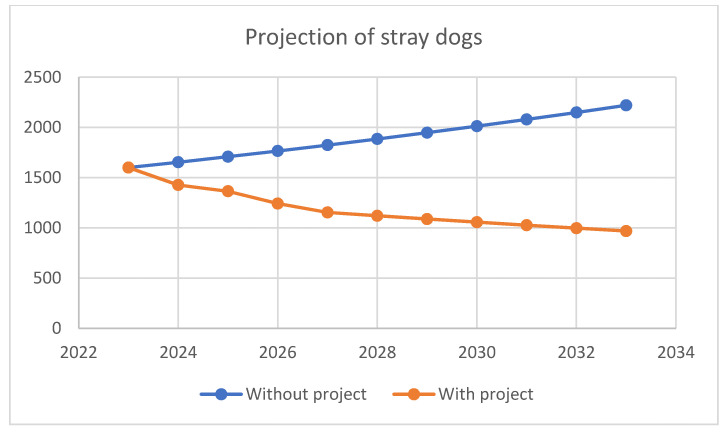
Projection of the number of stray dogs.

In order to increase the visibility for readers, Figure 3 presents forecasted trends of stray dogs with and without project.

The projection of total annual operating costs, in accordance with the above-defined assumptions, is presented in Table 9.

This shelter for dogs will provide funds to cover current operating costs, from revenues collected from municipalities, based on the number of dogs collected and processed from their territory.

The current data on the operation of the existing shelter for dogs in the municipality of Negotino and the prices this shelter charges for its services served as the basis for determining these revenues. Based on this price list, a complete 30-day treatment for stray dogs is charged at a price of EUR 125 per dog. Having in mind these data, but also a slightly shorter estimated period of stay for dogs in the shelter, a unit price of EUR 100 per stray dog was adopted for further calculations.

In addition to these basic incomes, the shelter can expect the realization of certain revenues based on the collection of owned dogs and their stay in the shelter. Namely, by counting the population of dogs in the field, it was determined that about 20% of owned dogs roam the streets. After the realization of the project, these dogs would also be collected and returned to their owners. This service would be charged at EUR 7.00 per day of stay, with an average estimated stay of 3 days. The project revenue projection is shown in Table 10.

The residual value of the project was estimated based on the following formula:

Y = (A/X) × (X − V)(1)

where Y—residual value of the project, A—value of investment, X—actual (physical) duration of the project, and V—analysis period.

In the continuation of this paper, we present the results of the financial analysis of this project. The financial analysis and evaluation of the project was performed using standard dynamic indicators of investment justification: Financial Net Present Value (FNPV), Financial Internal Rate of Return (FIRR), and Financial Benefit–Cost Ratio (FB/CR).

In order to reduce costs and benefits to the same base year, the discounting process was performed, so the prerequisite for a dynamic assessment of cost-effectiveness is the determination of an adequate discount rate. According to the recommendations of the European Commission [59], as already stated above, a recommended discount rate is 4% for countries acceding to the EU. Therefore, the effects of shelter construction—costs and benefits, which are annually considered in the period 2023–2033—are reduced to a common denominator by the selected discount rate, i.e., they are expressed in the current values of monetary units. This is shown in Table 11.

The results of the financial analysis, i.e., the indicators of investment justification, are shown in Table 12.

The financial net present value (FNPV) of this project is negative to the amount of EUR 75,291, which means that the company, if it expects a return at the rate of 4%, will be in profit for the amount of net present value. The Financial Internal Rate of Return (FIRR) of this project is 0.57%, which cannot be considered a favorable internal rate, given that the project is acceptable if the IRR is a minimum of 4%. The Financial Benefit–Cost Ratio (FB/CR) of this project is 0.925, which means that the value of total discounted revenues is lower than the value of total discounted costs.

As shown by the results of our analysis, and if not burdened with salary costs, the project does not provide satisfactory financial results. Therefore, the justification of projects of this type is primarily seen through the socio-economic benefits that such projects bring. However, it is important to point out that the shelter project for Vardar region can cover current costs from the realized income, which ensures its smooth functioning.

### 4.2. Economic Analysis

Economic CBA requires examining the impact of this project on the economic well-being of society. The purpose of economic analysis is to prove that the project has a positive contribution to society and is therefore worth implementing.

Project costs, considered in this analysis, are defined as the investment costs and operating costs of the maintenance and management of the new facility.

Investment costs were determined in the previous part of the paper and amount to EUR 366,251. Annual operating costs, in the initial year of project operation, are set at EUR 166,070. Operating costs increased in the observed period, in accordance with the estimate of Gross Domestic Product (GDP) growth in North Macedonia, but also decreased by a proportional part of the variable costs, due to the reduction in the number of dogs.

When conducting economic analysis, the economic prices of investments and costs are used, and the conversion of financial into economic prices is usually performed using sectoral conversion factors, if any. When sector-specific conversion factors are not available, the SCF is used based on the average differences between domestic and international prices due to trade tariffs and constraints, which can be estimated from foreign trade statistics using the following formula:

SCF = (M + X)/(M + X + Tm)(2)

where M—value of total imports, X—value of total exports, and Tm—value of customs revenues.

Based on the available data, the following calculation of the SCF was performed, as shown in Table 13.

The expected benefits from the realization of this project are multiple, because the adequate management of the stray dog population in a certain region has a number of significant benefits, making it both easier and harder to measure. Within this analysis, the following socio-economic benefits were quantified:

Savings in the cost caused by stray dog bites;
Savings in the cost of traffic accidents caused by stray dogs;
Cost savings in the treatment of diseases caused by stray dogs.

The realization of this project will reduce the number of stray dogs in the Vardar region, and thus reduce the possibility of a bite. These savings were calculated based on the following data: projections of the number of dogs in this region “without project” and “with project”, projections of bite cases by stray dogs “without project” and “with project”, and the determination of unit values of costs caused by stray dog bites.

Projections of cases of bites by stray dogs were determined on the basis of data from the previous period, obtained by certain municipalities in North Macedonia, as well as on the basis of the previously determined projection of the number of dogs in Vardar region. The data obtained refer to the municipalities of Veles, Negotino, and Demir Kapiju, so for other municipalities a proportional assessment was made in accordance with the individual area, in relation to these three municipalities.

Projections of the number of cases of stray dog bites “without project” and “with project” are shown in Table 14.

The cost of stray dog bites was calculated on the basis of data provided from North Macedonia on the value of lawsuits for bites. It is assumed that these values best provide insight into the value of these costs, because they include direct and indirect costs incurred in this way—the cost of treatment, the cost of pain and fear, the cost of absence from work, etc. Table 15 shows the reduction in the number of stray dog bites, as well as the savings in these costs.

**Figure 4:**
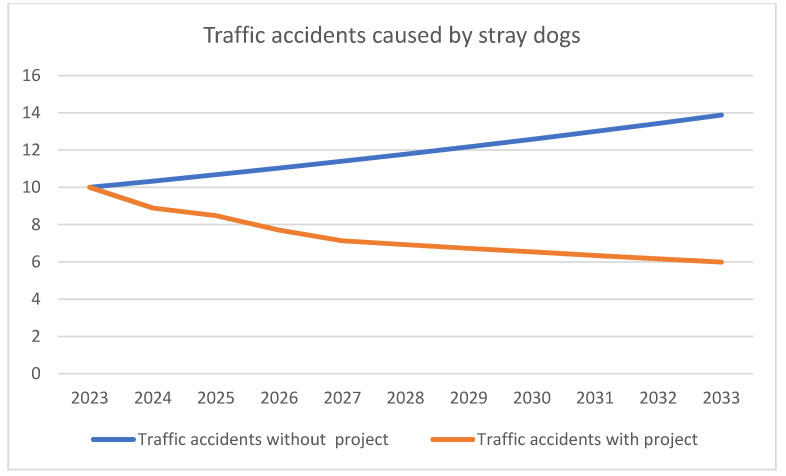
Projection of the number of traffic accidents caused by stray dogs.

In the continuation of the paper, we calculated savings on the cost of traffic accidents caused by stray dogs as the economic benefits of this project. In addition to the reduction in the number of bites by stray dogs, by reducing their number on the streets in this region, there will be a reduction in the number of traffic accidents, and thus savings in the cost of traffic accidents. These savings were calculated based on the following data: projections of the number of dogs in this region “without project” and “with project”, projections of traffic accidents caused by stray dogs “without project” and “with project”, structures of traffic accidents, and determined unit values of traffic accident costs. Projections of traffic accidents caused by stray dogs were determined on the basis of data from the previous period, provided by the Food and Veterinary Agency, Republic of North Macedonia, as well as on the basis of the previously determined projection of the number of dogs. These projections “without project” and “with project” are shown in Table 16 and Figure 4.

Due to the lack of specific data on the structure of traffic accidents caused by stray dogs in this region, we used the official data of the State Statistical Office of the Republic of North Macedonia, as shown in Table 17.

The unit costs of traffic accidents were determined as the average value of these costs on the basis of two models from the literature. The first model [63] defines these values for the EU28 average, and then proposes their adjustment for each country, in line with the GDP per capita. The second model [64] is based on a comprehensive analysis, which provides recommendations for determining the unit costs of accidents based on the established formula, in line with the GDP per capita. These values, as well as the results of the calculations, are shown in Table 18.

**Figure 5:**
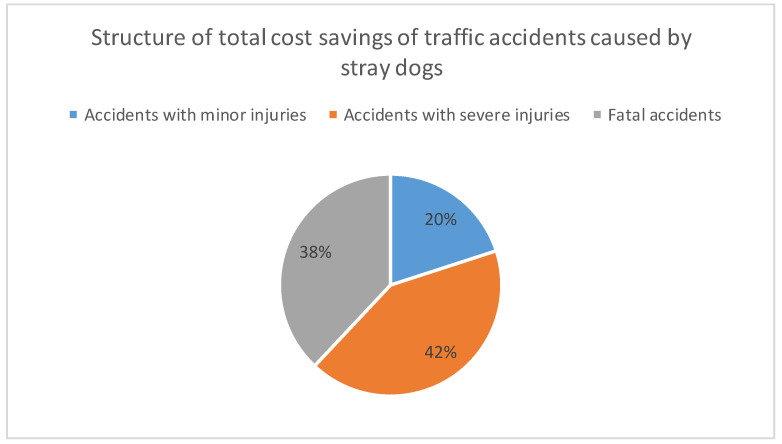
Structure of total cost savings for traffic accidents caused by stray dogs.

According to the previously determined values, savings in the costs of traffic accidents caused by stray dogs “without project” and “with project” were calculated, as presented in Table 19 and Figure 5.

In the continuation of this paper, we calculate the savings in the cost of treating diseases caused by stray dogs as significant additional economic benefits of this project. Stray dogs can affect the cause and spread of various diseases in humans and animals, and for the purposes of this study, we analyzed the effects of leishmaniasis and echinococcosis. The assumption is that with the realization of this project and the adequate health treatment of stray dogs, these diseases will be eradicated. Savings in the cost of treating diseases caused by stray dogs were calculated based on the following data: projections of the number of dogs in this region “without project”, projections of cases of disease caused by stray dogs “without project”, and determined unit values of disease treatment costs by stray dogs.

Projections of cases of disease caused by stray dogs (leishmaniasis and echinococcosis) were determined on the basis of data from the previous period, obtained by the Food and Veterinary Agency, Republic of North Macedonia, as well as on the basis of the previously determined projection of the number of dogs. Projections of the number of cases of diseases caused by stray dogs “without project” are shown in Table 20.

The unit values of the cost of treatment of leishmaniasis and echinococcosis were determined on the basis of data from the World Health Organization at the level of EUR 595 [65] for the case of leishmaniasis and EUR 2670 [66] for the case of echinococcosis. The cost savings for treating diseases caused by stray dogs are shown in Table 21.

Economic analysis and evaluation of this project was performed using standard dynamic indicators of investment justification: Net Present Value (NPV), Internal Rate of Return (IRR), and Benefit–Cost Ratio (B/CR).

In order to reduce costs and benefits to the same base year, the discounting process was performed, so the prerequisite for a dynamic assessment of cost-effectiveness is the determination of an adequate discount rate. According to the recommendations of the European Commission [59], as already stated above, a recommended discount rate is 5% for countries acceding to the EU. The effects of shelter construction—costs and benefits, which are annually considered in the period 2023–2033—are reduced to a common denominator by a selected discount rate, i.e., they are expressed in the current values of monetary units, as presented in Table 22.

**Figure 6:**
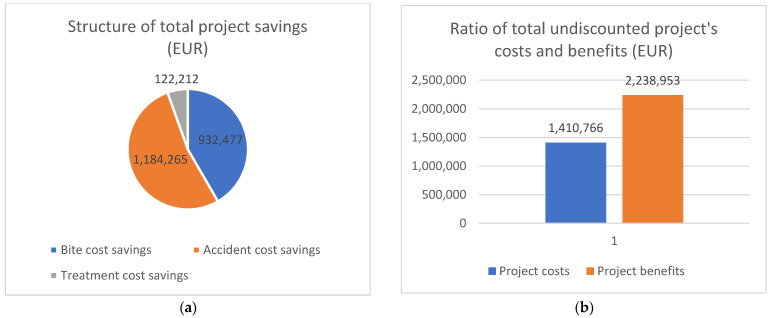
Structure of total project savings and ratio of total undiscounted costs and benefits. (**a**) Structure of total project savings; (**b**) ratio of total undiscounted costs and benefits of the project (EUR).

Based on the previous calculations, in the continuation of the paper, we present the structure of total project savings and the ratio of total undiscounted project costs and benefits, as shown in Figure 6.

In the following part, we present the final results. The results of the economic analysis, i.e., the indicators of investment justification, are shown in Table 23.

The Economic Net Present Value (ENPV) of this project is positive for an amount of EUR 789,916, which means that the company, if it expects a return at the rate of 5%, will be in profit for the amount of net present value. The Economic Internal Rate of Return (EIRR) of this project is 25.94%, which can be considered a favorable internal rate, given that the project is acceptable if the IRR is a minimum of 5%. The Economic Benefit–Cost Ratio (EB/CR) of this project is 1.90, which means that the value of the total discounted revenues is higher than the value of the total discounted costs.

Furthermore, we note that if the economic analysis of project justification includes salary costs, the project also remains in the zone of socio-economic justification (EIRR = 11.11%, ENPV = EUR 231,248, EB/CR = 1.16), which indisputably confirms the socio-economic justification for the implementation of this project.

### 4.3. Project Sensitivity Analysis

Considering that future values are used during the evaluation of project efficiency, which cause a certain greater or lesser degree of uncertainty in the obtained results, we performed sensitivity analysis for the project, which determined the project profitability threshold by varying the key analysis parameters, namely:

The volume of work of the shelter: ±10% and ±20%;
The size of the capital investment: +10% and +20%;
The discount rate: 6%, 8%, and 10.

A recapitulation of the results of the sensitivity analysis, with the assumptions made in the economic analysis, is given in Table 24.

From the aforementioned, it should be noted that the sensitivity analysis has shown that the project is resistant to all assumed real changes in input parameters, and that all indicators remain in the cost-effectiveness zone, which further strengthens the belief in the necessity and socio-economic justification of this project.

## 5. Conclusions

VPR in North Macedonia has long faced challenges related to the increasing number of stray dogs on the streets. Due to the insufficiency of the existing capacity, the need to build a new shelter that can meet the needs of this region has become actualized.

The financial analysis showed that for the realization of the shelter construction project, including its equipment, it is necessary to allocate financial resources in the amount of EUR 366,251. The indicators of financial feasibility of the project are below the profitability limit, which was expected for a project of this type, where the motives for implementation should be sought in terms of socio-economic rather than commercial effects. The expected economic benefits from the implementation of this project are, as already mentioned, multiple, and within the subject economic analysis, the following socio-economic benefits have been quantified: savings in stray dog bite costs, savings in the cost of traffic accidents caused by stray dogs, and cost savings in the treatment of diseases caused by stray dogs. The results of the analysis show that the investment in the construction of a stray dogs’ shelter has a satisfactory economic justification because the Economic Internal Rate of Return is higher than the opportunity cost of capital (EIRR = 25.94%), the Economic Net Present Value is greater than 0 (ENPV = 789,916 EUR), and the EB/CR (Economic Benefit–Cost Ratio) is greater than 1 (EB/CR = 1.90). Therefore, regarding the analyzed socio-economic effects of the realization of this project, we can unequivocally conclude that this investment has full socio-economic justification. The results of the conducted sensitivity analysis for this project further strengthen the belief that its implementation is necessary.

In future research, the model for financing the construction of the shelter will be specified, as well as the model for the management and operational functioning of the shelter. The need for this project is primarily focused in municipalities of the VPR, which are the initiators of its realization. For that reason, the basic and most probable model by which this project could be implemented is the establishment of a certain type of joint—a regional company, formed by local governments, whose rights, obligations, and competencies would be regulated by mutual agreement and relevant legal acts. In this regard, in financial terms, it is important to define two issues: sources of financing for the realization of planned investments, and the manner of covering future operating costs, i.e., achieving financial sustainability for the further functioning of the project. The first potential model of financing involves providing a donation for the implementation of the project—the construction of shelters. If a donation was not provided, municipalities in the VPR would have to jointly (in proportion to the number of inhabitants, the number of dogs, the area, the municipal budget, or in some other, jointly agreed way) provide the necessary funds for the construction of shelters, which is undoubtedly in their common interest. A second potential financing model could be a certain type of public–private partnership (PPP). One of the possibilities could be the joint financing of the investment (50% from the private investor and 50% from the municipality), after which the private partner would continue to manage the shelter, for the needs of the municipalities of the Vardar region.

We believe that the analysis carried out in our work represents a theoretical and practical contribution to this field, as well as to solving a specific problem. The method of quantifying and monetizing non-marketable, i.e., non-commercial, effects from the realization of this project represents the theoretical contribution of the work, which overcomes the imperfections of the evaluation of projects of this and similar character due to the quantification of only market effects.

Limitations in this paper may refer to the socio-economic feasibility study of the project, in which three types of socio-economic benefits were evaluated, being treated as cost savings. However, these are certainly not the only socio-economic effects of this project; the better availability of certain data would allow the other benefits to be quantified. This represents a free research space for other researchers and the improvement of similar research in the future.

The results of this analysis have practical implications that should help the decision-making process, supporting the stakeholders who will be involved in the implementation of this and similar projects, which represents a practical contribution of our paper. Additionally, we outline that only in combination with other control measures can the construction of shelters effectively solve the problems related to the population of stray dogs.

## Figures and Tables

**Table 1:** The municipalities of the VPR that are currently conducting population control through dog shelters and owners.

Settlement of the Shelter	Registration Number	Owner	Municipality in VPR Covered by Shelter
Kumanovo	KУ-ПK 002	VSC Todor Velkov Kumanovo	Sveti Nikole
Negotino	HE-ПK 003	Veterinary station Negotino	Negotino, Kavadarci, Rosoman
Kumanovo	KУ-ПK 008	Veterinary clinic, Ino Vet, Kumanovo	Demir Kapija
Čaška	BE-ПK 009	Giovski team	Veles

**Table 2:** Investment structure.

Type of Work	Amount (EUR)
Land costs ^1^	35,000
Facility construction costs ^2^	270,928
Vehicle purchase costs ^3^	20,000
Equipment procurement costs—shelter ^4^	6500
Costs of purchasing veterinary equipment ^5^	6730
Costs of planning and project documentation and costs of supervision ^6^	27,093
Total	366,251

^1^ Land costs are estimated at EUR 35,000, or EUR 7 per m^2^, for a future plot of about 5000 m^2^, on which the construction of a shelter is planned. ^2^ Facility construction costs include: construction work, craft works, unforeseen works, electrical works, mechanical works, plumbing, water system, water supply, sewerage system, and landscaping. ^3^ Vehicle procurement costs are estimated based on the current market prices of the corresponding vehicles for this purpose. Current prices vary, depending on the manufacturer, level of equipment, etc., so the purchase price of the vehicle is set at an average level of EUR 20,000. ^4^ Equipment procurement costs include procurement costs of: clothes, dog catching equipment, freezer, washing machine, computers, and office supplies. ^5^ Costs of purchasing veterinary equipment include the procurement costs of: surgical table, animal examination table, transport boxes, stainless steel boxes, surgical lamp, centrifuge, weighing scale, refrigerator, bar code reader, sterilizer, and microscope. ^6^ Costs of planning and project documentation and costs of supervision are estimated at 10% of the total construction costs, i.e., EUR 27,093.

**Table 3:** Service life of the equipment.

Type of Equipment	Service Life
Facility	40 years
Equipment of the first category ^1^	10 years
Equipment of the second category ^2^	5 years
Vehicle	5 years
Computer equipment	4 years
Special clothing and footwear	2 years

^1^ Equipment for catching dogs, surgical table, animal examination table, transport boxes, stainless steel boxes. ^2^ Freezer, washing machine, office supplies, surgical lamp, centrifuge, measuring scale, refrigerator, barcode reader, sterilizer, microscope.

**Table 4:** Structure of fixed operating costs.

Type of Costs	Amount (EUR)
Salary costs ^1^	76,500
Maintenance costs of the facility and cage ^2^	3200
Vehicle maintenance costs	2000
Utility costs (electricity, water, etc.)	1800
Marketing costs	600
Education costs	900
Insurance costs ^3^	810
Accounting costs ^4^	960
Costs of legal services ^5^	3000
Costs of training workers to take care of dogs ^6^	1200
Additional security costs ^7^	2500
Other costs	600
Total	94,070

^1^ The salaries of the employees of the shelter are calculated in the salary costs: director, one veterinarian (full time), one veterinarian only during the first year (part time), four workers to work with dogs, a cleaner, and a night guard. ^2^ The costs of cage maintenance include: costs of disinfection (0.30 EUR/m^2^, after each dog exit), disinfection (0.20 EUR/m^2^, 6 times a year), and rodent control (20 EUR per facility, two times a year). The maintenance costs of the facility itself are estimated at 0.5% of the investment value of the facility. ^3^ Insurance costs are estimated at 0.3% of the investment value of the facility. ^4^ According to the contract with the accounting agency for this type of service. ^5^ The costs of external legal services, according to the outsourcing system, are estimated at EUR 250 per month. ^6^ The costs of training workers to take care of dogs are calculated only in the first year at the amount of EUR 300 per worker. ^7^ Additional security costs are provided for the activity of providing shelter on weekends and holidays, according to the outsourcing system.

**Table 5:** Structure of variable operating costs.

Type of Costs	Amount (EUR)
Dog service costs ^1^	36,000
Veterinary clinic costs (regular—annual)	36,000
Total	72,000

^1^ The costs of serving the dogs were calculated at the price of EUR 30 per dog, while the costs of the veterinary clinic were calculated at the price of EUR 30 per dog. The costs of the veterinary clinic include: vaccination costs, EUR 10; sterilization costs, EUR 10; parasite treatment costs, EUR 3; and other surgical costs and treatment costs, EUR 7.

**Table 6:** Gross Domestic Product growth rate projection.

2022 ^1^	2023 ^2^	2024–2030 ^3^	2031–2033 ^4^
4%	3.7%	3.5%	3%

^1^ European Bank for Reconstruction and Development. ^2^ Ibid. ^3^ Statista. ^4^ Authors’ estimation.

**Table 7:** Projection of the number of dogs—without project.

Year	Number of Dogs	New Dogs	Dead Dogs	Adoption/Euthanasia
2023	1600	0	0	0
2024	1653	448	91	304
2025	1708	463	94	314
2026	1765	478	97	325
2027	1824	494	100	335
2028	1885	511	103	347
2029	1948	528	107	358
2030	2012	545	110	370
2031	2079	563	114	382
2032	2149	582	118	395
2033	2220	602	122	408

**Table 8:** Projection of the number of dogs—with project.

Year	Number of Dogs	New Dogs	Dead Dogs	Adoption/Euthanasia
2023	1600	0	0	0
2024	1426	236	90	320
2025	1364	210	87	185
2026	1242	201	78	246
2027	1153	183	73	199
2028	1120	219	68	185
2029	1088	213	66	179
2030	1056	207	64	174
2031	1026	201	62	169
2032	997	195	60	164
2033	968	189	59	159

**Table 9:** Projection of operating costs (EUR).

Year	Fixed Costs	Variable Costs	Operating Costs
2024	17,570	72,000	89,570
2025	16,922	64,220	81,142
2026	17,493	63,291	80,784
2027	18,084	59,617	77,701
2028	18,695	57,304	76,000
2029	19,328	57,602	76,930
2030	19,886	57,621	77,507
2031	20,461	57,640	78,101
2032	21,053	57,660	78,712
2033	21,662	57,679	79,342

**Table 10:** Project revenue projection (in EUR).

Year	Revenue from Dog Processing	Revenue from Owned Dogs
2024	120,000	5040
2025	110,724	4650
2026	105,919	4449
2027	96,397	4049
2028	89,524	3760
2029	86,945	3652
2030	84,033	3529
2031	81,613	3428
2032	79,263	3329
2033	76,980	3233

**Table 11:** Projection of the economic flow of the project—financial analysis (in EUR).

Year	Investment	Operational Costs	Revenue from Dog Processing	Revenue from Owned Dogs	Residual Value ^1^	Net Effects
2023	366,251					−366,251
2024	0	89,570	120,000	5040		35,470
2025	800	81,142	110,724	4650		33,433
2026	0	80,784	105,919	4449		29,584
2027	2700	77,701	96,397	4049		20,044
2028	24,930	76,000	89,524	3760		−7646
2029	800	76,930	86,945	3652		12,867
2030	0	77,507	84,033	3529		10,056
2031	2700	78,101	81,613	3428		4240
2032	0	78,712	79,623	3329		3880
2033	0	79,341	76,980	3233	239,146	240,018

^1^ The residual value was positioned in the last year of the analysis (2033) within the economic flow projection, and then discounted to the present value when calculating the net effects. Based on the stated total value of the investment and the service life of the facility and equipment, the residual value was calculated in the amount of EUR 239,146 and was included in the economic flow of the project in the last observed year.

**Table 12:** Review of project financial feasibility indicators.

Feasibility Indicators	Value
Financial Net Present Value (FNPV)	EUR −75,291
Financial Internal Rate of Return (FIRR)	0.57%
Financial Benefit–Cost Ratio (FB/CR)	0.925

**Table 13:** Calculation of standard conversion factor (SCF).

Description	Amount (in USD Million)
Total imports	9446 ^1^
Total exports	7198 ^1^
Customs revenue	1631 ^2^
Standard conversion factor (SCF)	0.91

^1^ State Statistical Office, Republic of North Macedonia, data for 2019, https://www.stat.gov.mk/ (accessed on 25 August 2022). ^2^ Customs Administration, Republic of North Macedonia, https://customs.gov.mk/index.php/mk/ (accessed on 25 August 2022).

**Table 14:** Projection of the number of cases of bites by stray dogs.

Year	Number of Dogs “without Project”	Number of Bites “without Project”	Number of Dogs “with Project”	Number of Bites “with Project”
2023	1600	151	1600	151
2024	1653	156	1426	135
2025	1708	161	1364	129
2026	1765	167	1242	117
2027	1824	172	1153	109
2028	1885	178	1120	106
2029	1948	184	1088	103
2030	2012	190	1056	100
2031	2079	196	1026	97
2032	2149	203	997	94
2033	2220	210	968	91

**Table 15:** Savings on the cost of stray dog bites.

Year	Number of Bites “without Project”	Number of Bites “with Project”	Decrease in Number of Bites	Savings from Costs of Bites ^1^
2024	156	135	21	26,765
2025	161	129	32	40,562
2026	167	117	49	61,744
2027	172	109	63	79,124
2028	178	106	72	90,208
2029	184	103	81	101,417
2030	190	100	90	112,763
2031	196	97	99	124,258
2032	203	94	109	135,912
2033	210	91	118	147,739

^1^ The costs of stray dog bites vary, so for the further analysis and determination of the savings in these costs, we used an average value of EUR 1250 per case. The stated value was obtained on the basis of data from the municipality of Veles (average value of compensation of EUR 1135 for 67 bite cases) and the municipality of Negotino (average value of compensation of EUR 2000 for 10 bite cases). The weighting procedure reduced these values to the proposed average value of EUR 1250 per case.

**Table 16:** Projections of the number of cases of traffic accidents caused by stray dogs.

Year	Number of Dogs “without Project”	Number of Traffic Accidents “without Project”	Number of Dogs “with Project”	Number of Traffic Accidents “with Project”
2023	1600	10	1600	10
2024	1653	10	1426	9
2025	1708	11	1364	9
2026	1765	11	1242	8
2027	1824	11	1153	7
2028	1885	12	1120	7
2029	1948	12	1088	7
2030	2012	13	1056	7
2031	2079	13	1026	6
2032	2149	13	997	6
2033	2220	14	968	6

**Table 17:** Structure of traffic accidents in the Republic of North Macedonia, 2019.

Structure of Traffic Accidents ^1^	Number of Accidents	Participation
Fatal accidents	148	2.5%
Accidents with severe injuries	839	13.8%
Accidents with minor injuries	5074	83.7%
Total accidents	6061	100.0%

^1^ State Statistical Office, Republic of North Macedonia, Transport and Communication, https://www.stat.gov.mk/OblastOpsto.aspx?id=24 (accessed on 12 August 2022).

**Table 18:** Unit costs of traffic accidents (EUR).

Structure of Traffic Accidents	IRAP ^1^	EC Handbook ^2^	Adopted Values
Fatal accidents	324,360	425,859	375,110
Accidents with severe injuries	81,090	64,855	72,973
Accidents with minor injuries	6244	5010	5627

^1^ International Road Assessment Program [63]. ^2^ European Commission [64].

**Table 19:** Savings in the cost of traffic accidents caused by stray dogs (in EUR).

Year	Accidents with Minor Injuries	Accidents with Severe Injuries	Fatal Accidents	Savings from Traffic Accident Costs
2024	6680	14,324	12,988	33,992
2025	10,123	21,708	19,684	51,515
2026	15,410	33,044	29,963	78,417
2027	19,747	42,345	38,397	100,489
2028	22,513	48,277	43,776	114,565
2029	25,311	54,275	49,215	128,802
2030	28,143	60,348	54,721	143,212
2031	31,011	66,499	60,299	157,810
2032	33,920	72,736	65,955	172,612
2033	36,871	79,065	71,694	187,631

**Table 20:** Projections of the number of cases of disease caused by stray dogs—without the realization of the project.

Year	Number of Dogs “Without Project”	Number of Leishmaniasis Cases	Number of Echinococcosis Cases
2024	1653	0.26	3.87
2025	1708	0.27	4.00
2026	1765	0.28	4.14
2027	1824	0.29	4.28
2028	1885	0.29	4.42
2029	1948	0.30	4.56
2030	2012	0.31	4.72
2031	2079	0.32	4.87
2032	2149	0.34	5.04
2033	21,662	0.35	5.20

**Table 21:** Projection of cost savings for the treatment of diseases caused by stray dogs (in EUR).

Year	Leishmaniasis Costs	Echinococcosis Costs	Treatment Savings Costs
2024	154	10,346	10,500
2025	159	10,690	10,849
2026	164	11,046	11,211
2027	170	11,414	11,584
2028	175	11,794	11,970
2029	181	12,187	12,368
2030	187	12,593	12,780
2031	193	13,012	13,206
2032	200	13,446	13,645
2033	206	13,893	14,100

**Table 22:** Projection of economic flow of the project—economic analysis (in EUR).

Year	Investment	Operational Costs	Bite Cost Savings	Accident Cost Savings	Treatment Cost Savings	Residual Value	Net Effects
2023	333,288						−333,288
2024	0	81,509	26,765	33,992	10,500		−10,253
2025	728	73,839	40,562	51,515	10,849		28,359
2026	0	73,513	61,744	78,417	11,211		77,858
2027	2457	70,708	79,124	100,489	11,584		118,032
2028	22,686	69,160	90,208	114,565	11,970		124,897
2029	728	70,006	101,417	128,802	12,368		171,953
2030	0	70,531	112,763	143,212	12,780		198,224
2031	2457	71,072	124,258	157,810	13,206		221,745
2032	0	71,628	135,912	172,612	13,645		250,541
2033	0	72,200	147,739	187,631	14,100	217,623	494,892

**Table 23:** Review of project economic feasibility indicators.

Feasibility Indicators	Value
Economic Net Present Value (ENPV)	EUR 789,916
Economic Internal Rate of Return (EIRR)	25.94%
Economic Benefit–Cost Ratio (FB/CR)	1.90

**Table 24:** Recapitulation of sensitivity analysis results.

No.	Type of Test	EIRR Condition: EIRR > OCC	ENPV Condition: ENPV > 0	EB/CR Condition: EB/CR > 1
1.	SCOPE OF WORK			
	Scenario 1: Base scenario	25.94%	789,916	1.90
	Scenario 2: Cost savings 10% smaller	22.42%	636,275	1.73
	Scenario 3: Cost savings 20% smaller	18.70%	482,642	1.55
	Scenario 4: Cost savings 10% higher	29.30%	943,554	2.08
	Scenario 5: Cost savings 20% higher	32.52%	1,097,191	2.26
2.	INVESTMENT COST			
	Scenario 1: Base scenario	25.94%	789,916	1.90
	Scenario 2: Investment growth by 10%	24.16%	768,740	1.85
	Scenario 3: Investment growth by 20%	22.60%	747,564	1.79
3.	DISCOUNT RATE			
	Scenario 1: Base scenario—discount rate 5%	25.94%	789,916	1.90
	Scenario 2: Discount rate 6%	25.94%	708,696	1.84
	Scenario 3: Discount rate 8%	25.94%	567,983	1.73
	Scenario 4: Discount rate 10%	25.94%	451,565	1.62

## Data Availability

Not applicable.
